# Supplementary material for: Recognition of Long-COVID-19 Patients in a Canadian Tertiary Hospital Setting: A Retrospective Analysis of Their Clinical and Laboratory Characteristics
Source: Pathogens. 2021 Sep 26;10(10):1246. doi: 10.3390/pathogens10101246 (PMC8537802; doi:10.3390/pathogens10101246)
Supplement: Supplementary file 1 [file pathogens-10-01246-s001.zip › pathogens-1353051-supplementary.pdf]

pathogens

MDPI

Supplemental Table S1: Biochemistry (+/- 7 days)

| | LONG COVID GROUP, n = 62 | | | | | | NON-LONG COVID GROUP, n = 161 | | | | | | p-value |
|---|---|---|---|---|---|---|---|---|---|---|---|---|---|
| | Total | A | B | C | D | E | Total | A | B | C | D | E | |
| **Laboratory Investigations** | | | | | | | | | | | | | |
| **Number (patients)** | n = 31 | -- | n = 9 | n = 3 | n = 9 | n = 10 | n = 65 | -- | n = 28 | n = 18 | n = 14 | n = 5 | |
| **Hemoglobin, mean (SD)** | 133.5 (17.3) | -- | 139.7 (8.2) | 130.3 (9.0) | 140.2 (12.0) | 123.0 (22.9) | 134.9 (18.8) | -- | 142.7 (18.0) | 128.3 (16.2) | 127.6 (20.6) | 135.0 (2.8) | 0.7404 |
| **[confidence interval]** | 127.5 to 139.6 | -- | 134.3 to 145.0 | 120.2 to 140.5 | 132.4 to 148.0 | 108.8 to 137.2 | 130.3 to 139.5 | -- | 136.0 to 149.4 | 120.9 to 135.8 | 116.8 to 138.4 | 132.6 to 137.4 | |
| **Time from COVID+ (days)** | 0.19 (1.4) | -- | 0.0 (0.0) | -1.0 (0.82) | -0.11 (0.31) | 1.0 (2.1) | 0.42 (2.2) | -- | 0.18 (2.2) | 0.28 (2.2) | 0.43 (1.9) | 2.2 (2.7) | 0.5570 |
| **mean (SD) {range}** | (-2) to 6 | -- | 0 - 0 | (-2) - 0 | (-1) - 0 | (-1) - 6 | (-6) - 6 | -- | (-5) - 6 | (-6) - 6 | (-4) - 5 | 0 - 6 | |
| **[confidence interval]** | (-0.29) to 0.68 | -- | -- | (-1.9) to (-0.08) | (-0.32) to 0.09 | (-0.30) to 2.3 | (-0.13) to 0.96 | -- | (-0.64) to 1.0 | (-0.72) to 1.3 | (-0.58) to 1.4 | (-0.18) to 4.6 | |
| **Number (patients)** | n = 31 | -- | n = 9 | n = 3 | n = 9 | n = 10 | n = 65 | -- | n = 28 | n = 18 | n = 14 | n = 5 | |
| **Platelets, mean (sd)** | 202.8 (66.3) | -- | 200.0 (46.3) | 303 (78.5) | 199.8 (64.6) | 177.9 (49.5) | 194.4 (73.5) | -- | 185.4 (74.8) | 213.7 (86.5) | 197.8 (50.9) | 166.4 (46.8) | 0.5963 |
| **[confidence int]** | 179.4 to 226.1 | -- | 169.7 to 230.3 | 214.2 to 391.8 | 157.6 to 242.0 | 147.2 to 208.6 | 176.5 to 212.3 | -- | 157.7 to 213.1 | 173.7 to 253.6 | 171.1 to 224.4 | 125.3 (207.5) | |
| **Time from COVID+ (days)** | 0.19 (1.4) | -- | 0.0 (0.0) | -1.0 (0.82) | -0.11 (0.31) | 1.0 (2.1) | 0.42 (2.2) | -- | 0.18 (2.2) | 0.28 (2.2) | 0.43 (1.9) | 2.2 (2.7) | 0.5570 |

| | | | | | | | | | | | | | |
|---|---|---|---|---|---|---|---|---|---|---|---|---|---|
| **Mean (SD) {range} [confidence int]** | (-2) to 6 | -- | 0 - 0 | (-2) - 0 | (-1) - 0 | (-1) - 6 | (-6) - 6 | -- | (-5) - 6 | (-6) - 6 | (-4) - 5 | 0 - 6 | |
| | (-0.29) to 0.68 | -- | -- | (-1.9) to (-0.08) | (-0.32) to 0.09 | (-0.30) to 2.3 | (-0.13) to 0.96 | -- | (-0.64) to 1.0 | (-0.72) to 1.3 | (-0.58) to 1.4 | (-0.18) to 4.6 | |
| **Number (patients)** | n = 22 | -- | n = 5 | n = 3 | n = 5 | n = 9 | n = 34 | -- | n = 9 | n = 11 | n = 10 | n = 4 | |
| **PT (INR) Mean (sd) [confidence int]** | 12.95 (1.3) | -- | 12.1 (0.48) | 12.4 (0.64) | 13.1 (1.4) | 13.5 (1.5) | 12.8 (1.0) | -- | 12.4 (0.89) | 12.4 (0.71) | 13.1 (1.2) | 13.7 (0.94) | 0.5629 |
| | 12.4 to 13.5 | -- | 11.7 to 12.6 | 11.7 to 13.1 | 11.9 to 14.3 | 12.5 to 14.5 | 12.4 to 13.1 | -- | 11.8 to 13.0 | 12.0 to 12.9 | 12.4 to 13.8 | 12.8 to 14.6 | |
| **Time from COVID+ (days) Mean (SD) {range} [confidence int]** | 0.41 (2.1) | -- | 0.0 (0.0) | -2.0 (1.6) | -0.2 (1.6) | 1.8 (2.1) | 0.76 (2.2) | -- | 1.4 (1.8) | 0.36 (1.9) | 0.5 (2.5) | 1.0 (2.9) | 0.5602 |
| | (-4) - 6 | -- | 0 - 0 | (-4) - 0 | (-3) - 2 | (-1) - 6 | (-4) - 6 | -- | (-1) - 4 | (-1) - 6 | (-4) - 5 | (-1) - 6 | |
| | (-0.47) to 1.3 | -- | -- | (-3.9) to (-0.15) | (-1.6) to 1.2 | 0.41 to 3.1 | 0.02 to 1.5 | -- | 0.29 to 2.6 | (-0.74) to 1.5 | (-1.0) to 2.0 | (-1.9) to 3.9 | |
| **Number (patients)** | n = 18 | -- | n = 4 | n = 0 | n = 5 | n = 9 | n = 15 | -- | n = 1 | n = 5 | n = 5 | n = 4 | |
| **PTT Mean (sd) [confidence int]** | 27.6 (4.4) | -- | 24.4 (1.5) | -- | 24.8 (1.1) | 30.6 (4.3) | 24.7 (3.0) | -- | 24.7 (0.0) | 22.4 (1.5) | 25.3 (3.4) | 26.9 (1.9) | 0.0417* |
| | 25.6 to 29.6 | -- | 23.0 to 25.9 | -- | 23.8 to 25.7 | 27.9 to 33.4 | 23.2 to 26.2 | -- | -- | 21.1 to 23.7 | 22.3 to 28.3 | 25.1 to 28.8 | |
| **Time from COVID+ (days) Mean (SD) {range} [confidence int]** | 0.78 (1.99) | -- | 0.0 (0.0) | -- | -0.2 (1.6) | 1.7 (2.2) | -0.07 (2.2) | -- | 0.0 (0.0) | -0.2 (0.75) | -1.4 (2.2) | 1.8 (2.5) | 0.2711 |
| | (-3) - 6 | -- | 0 - 0 | -- | (-3) - 2 | (-1) - 6 | (-4) - 6 | -- | 0 - 0 | (-1) - 0 | (-4) - 1 | 0 - 6 | |
| | (-0.14) to 1.7 | -- | -- | -- | (-1.6) to 1.2 | 0.22 to 3.1 | (-1.2) to 1.0 | -- | -- | (-0.86) to 0.46 | (-3.3) to 0.49 | (-0.69) to 4.2 | |

| | | | | | | | | | | | | | |
|---|---|---|---|---|---|---|---|---|---|---|---|---|---|
| **Number (patients)** | n = 31 | -- | n = 9 | n = 3 | n = 9 | n = 10 | n = 65 | -- | n = 28 | n = 18 | n = 14 | n = 5 | |
| **Lymphocytes Mean (SD)** | 1.0 (0.53) | -- | 1.1 (0.57) | 1.3 (0.45) | 1.2 (0.48) | 0.83 (0.46) | 1.1 (0.54) | -- | 1.2 (0.61) | 1.1 (0.46) | 1.1 (0.55) | 0.86 (0.23) | 0.5451 |
| **[confidence int]** | 0.85 to 1.22 | -- | 0.70 to 1.5 | 0.76 to 1.8 | 0.84 to 1.5 | 0.54 to 1.1 | 0.98 to 1.2 | -- | 0.97 to 1.4 | 0.84 to 1.3 | 0.82 to 1.4 | 0.66 to 1.1 | |
| **Lymphocytes % Mean (SD)** | 17.5 (10.3) | -- | 22.1 (13.9) | 17.8 (6.3) | 16.8 (7.3) | 13.9 (7.9) | 19.9 (11.4) | -- | 23.0 (11.7) | 18.6 (10.7) | 17.7 (10.9) | 13.4 (7.3) | 0.3317 |
| **[confidence int]** | 13.9 to 21.1 | -- | 13.1 to 31.2 | 10.7 to 24.9 | 12.1 to 21.6 | 9.1 to 18.8 | 17.1 to 22.7 | -- | 18.7 to 27.3 | 13.7 to 23.5 | 12.0 to 23.3 | 7.0 to 19.8 | |
| **Monocytes Mean (SD)** | 0.45 (0.23) | -- | 0.51 (0.18) | 0.57 (0.39) | 0.37 (0.19) | 0.42 (0.21) | 0.54 (0.23) | -- | 0.45 (0.18) | 0.61 (0.27) | 0.61 (0.21) | 0.58 (0.22) | 0.0634 |
| **[confidence int]** | 0.36 to 0.53 | -- | 0.39 to 0.63 | 0.13 to 1.0 | 0.24 to 0.49 | 0.29 to 0.55 | 0.49 to 0.60 | -- | 0.39 to 0.52 | 0.49 to 0.74 | 0.51 to 0.72 | 0.38 to 0.78 | |
| **Monocytes % Mean (SD)** | 7.4 (3.8) | -- | 10.0 (3.0) | 6.9 (3.5) | 5.3 (2.1) | 7.1 (4.2) | 8.9 (3.7) | -- | 8.7 (3.9) | 9.9 (4.0) | 8.9 (2.6) | 7.2 (2.0) | 0.0644 |
| **[confidence int]** | 6.1 to 8.7 | -- | 8.1 to 12.0 | 2.9 to 10.9 | 3.9 to 6.7 | 4.4 to 9.7 | 8.1 to 9.8 | -- | 7.2 to 10.1 | 8.0 to 11.7 | 7.5 to 10.3 | 5.5 to 9.0 | |
| **Neutrophils Mean (SD)** | 5.1 (2.7) | -- | 3.8 (2.4) | 5.6 (1.8) | 6.1 (3.3) | 5.2 (2.0) | 4.7 (2.4) | -- | 4.0 (1.9) | 5.0 (2.8) | 5.2 (2.1) | 6.5 (3.1) | 0.5199 |
| **[confidence int]** | 4.1 to 6.0 | -- | 2.3 to 5.4 | 3.6 to 7.6 | 4.0 to 8.2 | 3.9 to 6.4 | 4.1 to 5.3 | -- | 3.3 to 4.6 | 3.7 to 6.3 | 4.1 to 6.3 | 3.8 to 9.2 | |
| **Neutrophils % Mean (SD)** | 74.4 (11.6) | -- | 66.9 (14.1) | 75 (2.9) | 77.3 (7.6) | 78.3 (10.3) | 70.1 (13.8) | -- | 67.2 (14.3) | 70.3 (14.1) | 72.6 (12.3) | 78.6 (7.3) | 0.1422 |
| **[confidence int]** | 70.3 to 78.5 | -- | 57.7 to 76.1 | 71.7 to 78.3 | 72.4 to 82.3 | 71.9 to 84.7 | 66.8 to 73.5 | -- | 61.9 to 72.5 | 63.8 to 76.8 | 66.2 to 79.1 | 72.2 to 85.0 | |
| **WBC Count Mean (SD)** | 6.6 (2.8) | -- | 5.5 (2.4) | 7.4 (2.1) | 7.7 (3.6) | 6.5 (1.8) | 6.4 (2.5) | -- | 5.7 (2.2) | 6.8 (2.7) | 7.0 (1.9) | 8.0 (3.1) | 0.7832 |
| **[confidence int]** | 5.6 to 7.6 | -- | 3.9 to 7.0 | 5.0 to 9.8 | 5.3 to 10.0 | 5.3 to 7.6 | 5.8 to 7.1 | -- | 4.9 to 6.5 | 5.5 to 8.0 | 6.0 to 8.0 | 5.2 to 10.7 | |

| | | | | | | | | | | | | | |
|---|---|---|---|---|---|---|---|---|---|---|---|---|---|
| **Time from COVID+ (days)** | 0.19 (1.4) | -- | 0.0 (0.0) | -1.0 (0.82) | -0.11 (0.31) | 1.0 (2.1) | 0.42 (2.2) | -- | 0.18 (2.2) | 0.28 (2.2) | 0.43 (1.9) | 2.2 (2.7) | 0.5570 |
| **Mean (SD)** | (-2) - 6 | -- | 0 - 0 | (-2) - 0 | (-1) - 0 | (-1) - 6 | (-6) to 6 | -- | (-5) to 6 | (-6) - 6 | (-4) - 5 | 0 - 6 | |
| **{range} [confidence int]** | (-0.29) to 0.68 | -- | -- | (-1.9) to (-0.08) | (-0.32) to 0.09 | (-0.30) to 2.3 | (-0.13) to 0.96 | -- | (-0.64) to 1.0 | (-0.72) to 1.3 | (-0.58) to 1.4 | (-0.18) to 4.6 | |
| **Number (patients)** | n = 31 | -- | n = 9 | n = 3 | n = 9 | n = 10 | n = 64 | -- | n = 27 | n = 18 | n = 14 | n = 5 | |
| **Creatinine, mean (sd)** | 112.0 (130.5) | -- | 78.4 (17.0) | 81.7 (12.7) | 95.9 (59.5) | 165.7 (211.8) | 124.0 (181.3) | -- | 105.9 (122.6) | 101.2 (58.1) | 180.4 (330.0) | 146.0 (97.0) | 0.7448 |
| **[confidence int]** | 66.0 to 157.9 | -- | 67.4 to 89.5 | 67.3 to 96.0 | 57.0 to 134.8 | 34.4 to 297.0 | 79.6 to 168.4 | -- | 59.6 to 152.1 | 74.3 to 128.0 | 7.5 to 353.2 | 61.0 to 231.0 | |
| **Time from COVID+ (days)** | 0.29 (1.3) | -- | 0.0 (0.0) | 0.0 (0.82) | -0.11 (0.31) | 1.0 (2.1) | 0.33 (2.2) | -- | 0.0 (1.9) | 0.28 (2.2) | 0.43 (1.9) | 2.0 (2.9) | 0.9173 |
| **Mean (SD)** | (-1) - 6 | -- | 0 - 0 | (-1) - 1 | (-1) to 0 | (-1) - 6 | (-6) - 6 | -- | (-5) - 4 | (-6) - 6 | (-4) - 5 | (-1) - 6 | |
| **{range} [confidence int]** | (-0.18) to 0.76 | -- | -- | (-0.92) to 0.92 | (-0.32) to 0.09 | (-0.30) to 2.3 | (-0.20) to 0.86 | -- | (-0.73) to 0.733 | (-0.72) to 1.3 | (-0.58) to 1.4 | (-0.54) to 4.5 | |
| **Number (patients)** | n = 10 | -- | n = 2 | n = 1 | n = 3 | n = 4 | n = 14 | -- | n = 5 | n = 2 | n = 3 | n = 4 | |
| **D-dimer, Mean (sd)** | 795.1 (442.7) | -- | 550.5 (290.5) | 309 (0.0) | 897 (344.1) | 962.5 (483.8) | 1003 (865.4) | -- | 685.6 (564.0) | 980.5 (350.5) | 635.7 (194.5) | 1686.5 (1199.7) | 0.5113 |
| **[confidence int]** | 520.7 to 1069.5 | -- | 147.9 to 953.1 | 309 - 309 | 507.6 to 1286.4 | 488.4 to 1436.6 | 549.7 to 1456.3 | -- | 191.2 to 1180.0 | 494.7 to 1466.3 | 415.6 to 855.8 | 510.8 to 2862.2 | |
| **Time from** | 1.7 (2.2) | -- | 0.0 (0.0) | 0.0 (0.0) | 1.0 (1.4) | 3.5 (2.3) | 2.3 (2.4) | -- | 1.8 (1.7) | -0.5 (0.5) | 2.3 (2.5) | 4.3 (1.9) | 0.5679 |
| **COVID+ (days)** | 0 - 7 | -- | 0 - 0 | 0 - 0 | 0 - 3 | 1 - 7 | (-1) - 7 | -- | 0 - 5 | (-1) - 0 | (-1) - 5 | 2 - 7 | |
| **Mean (SD) {range} [confidence int]** | 0.31 to 3.1 | -- | -- | -- | (-0.60) to 2.6 | 1.3 to 5.7 | 1.0 to 3.5 | -- | 0.29 to 3.3 | (-1.2) to 0.19 | (-0.49) to 5.2 | 2.4 to 6.1 | |

*Values shown are mean (standard deviation), range if applicable and confidence interval with $\alpha = 0.05$. Statistical tests used were unpaired t-tests, unpaired t-tests with Welch's corrections and Fisher's exact test for comparing proportions.*

*Number (patients) denotes number of patients with relevant results recorded and used for analysis.*

*PT-INR = Prothrombin Time and International Normalized Ratio. PTT = Partial Thromboplastin Time. WBC Count = White Blood Cell Count. Time from COVID+ = time in days between the patients first positive COVID-19 diagnosis and the specific laboratory result.*
